# Influence of Previous General Anesthesia on Cognitive Impairment: An Observational Study Among 151 Patients

**DOI:** 10.3389/fnhum.2022.810046

**Published:** 2022-05-16

**Authors:** Federico Linassi, Alessandro De Laurenzis, Eleonora Maran, Alessandra Gadaldi, Leonardo Spano', Gino Gerosa, Demetrio Pittarello, Paolo Zanatta, Michele Carron

**Affiliations:** ^1^Department of Pharmaceutical and Pharmacological Sciences, University of Padova, Padova, Italy; ^2^Department of Medicine, Anaesthesiology and Intensive Care, University of Padova, Padova, Italy; ^3^Department of Cardiac, Thoracic, Vascular Sciences and Public Health, University of Padova, Padova, Italy; ^4^Department of Anesthesia and Intensive Care, Integrated University Hospital of Verona, Verona, Italy

**Keywords:** general anesthesia, age, cardiac surgery, comorbidities, pre-operative neurocognitive disorder

## Abstract

**Introduction:**

Preoperative neurocognitive disorder (preO-NCD) is a common condition affecting 14–51. 7% of the elderly population. General anesthesia has already been associated with the one-year post-operative neurocognitive disorder (PostO-NCD), specifically, a deficit in executive function, measured by the Trail Making Test B (TMT-B), but its long-term effects on cognitive function have not been investigated. We aimed to detect preO-NCD prevalence in patients scheduled for cardiac surgery and further investigate the possible role of previous general anesthesia (pGA) in general preoperative cognitive status [measured *via* the Montreal Cognitive Assessment (MoCA)] and/or in executive functioning (measured *via* TMT-B).

**Methods:**

In this observational, prospective study, 151 adult patients scheduled for elective cardiac surgery underwent MoCA and TMT-B. Data on age, education, pGA, comorbidities, and laboratory results were collected.

**Results:**

We discovered a general cognitive function impairment of 79.5% and an executive function impairment of 22%. Aging is associated with an increased likelihood (OR 2.99, *p* = 0.047) and education with a decreased likelihood (OR 0.35, *p* = 0.0045) of general cognitive impairment, but only education was significantly associated with a decreased likelihood (OR 0.22, *p* = 0.021) of executive function impairment. While pGA did not significantly affect preO-NCD, a noteworthy interaction between aging and pGA was found, resulting in a synergistic effect, increasing the likelihood of executive function impairment (OR 9.740, *p* = 0.0174).

**Conclusion:**

We found a high prevalence of preO-NCD in patients scheduled for cardiac surgery. General cognitive function impairment is highly associated with advancing age (not pGA). However, older patients with at least one pGA appeared to be at an increased risk of preO-NCD, especially executive function impairment, suggesting that TMT-B should be associated with MoCA in the preoperative cognitive evaluation in this population.

## Introduction

Mild cognitive impairment (MCI) refers to a range of conditions that occur frequently in the general population; it is estimated that 14–51.7% (Scott et al., 2018) of elderly people suffer from MCI and that an additional 10% suffer from a major cognitive disorder or dementia. MCI is described as a mild cognitive and functional impairment that is greater than expected for a patient's age and education level but is not severe enough to warrant a diagnosis of dementia (DSM-5 defines MCI as a “mild neurocognitive disorder”), thus representing part of a declining cognitive trajectory between normal aging and overt dementia (Lonie et al., 2009; Crosby et al., 2011).

Individuals with MCI are at a greater risk of progressing to major cognitive disorders; however, studies have shown that up to 25% of patients with MCI can revert to normal cognition (Malek-Ahmadi, 2016). This makes MCI an important temporal “window” in which intervening or delaying progression to dementia may be possible (Anderson, 2019). The core clinical criteria for the diagnosis of MCI are as follows: concern about a change in cognition expressed by the patient or an informant, impairment in one or more cognitive domains, the preservation of independence in functional abilities, and absence of dementia (Albert et al., 2011).

The Montreal Cognitive Assessment (MoCA), a test developed specifically for the detection of MCI (Nasreddine et al., 2005), has a sensitivity of 80–100% and a specificity of 50–76% using a cut-off point of 25 (Lin et al., 2013) to make a diagnosis, and it has been shown to be more accurate than Mini Mental Status Evaluation (MMSE) in accurately differentiating individuals with MCI from those with normal cognition (McKhann et al., 2011).

In addition to the MoCA, various neuropsychological tests can be administered for the diagnosis of MCI; among others, the Trail Making Test (TMT) is one of the most popular neuropsychological tests that provides information on visual search, scanning, speed of processing, mental flexibility, and executive function. It has been shown to be highly sensitive in detecting anesthesia-related impairment and post-operative neurocognitive disorder (PostO-NCD; Reitan, 1955; Sánchez-Cubillo et al., 2009). The TMT consists of two parts, Part A and Part B; measures of rote memory, visual scanning, graphomotor speed, and visuomotor processing speed were more related to the performance of the TMT-A score, while working memory and inhibition control were mainly associated with the TMT-B, which is an indicator of executive functioning (Arbuthnott and Frank, 2000; Llinàs-Reglà et al., 2017).

The etiology of MCI is complex, and the roles of multiple biological, social, and environmental factors have been investigated. Although they are non-modifiable, age, sex, family history of dementia, and genetics are considered to be the most important risk factors (Deckers et al., 2015). To develop more effective preventative and treatment interventions, researchers have paid increased attention to modifiable environmental and lifestyle risk factors; among these, lower educational level, vascular disease, obesity, and sleep-disordered breathing seem to have a major impact (Plassman et al., 2008; Iwashyna et al., 2010; Petersen et al., 2010; Yaffe et al., 2011).

Other potential risk factors for MCI, especially in the population with high cardiovascular comorbidity risk factors, are previous transitory ischaemic attacks (TIAs; Nicolas et al., 2020): one-third of patients with TIA have a profile similar to that of vascular MCI (van Rooij et al., 2016).

The influence of anesthesia and surgery on cognitive functions has been extensively studied. Data from animal experiments, in particular, suggest that anesthetic exposure causes neuronal apoptosis and impairs normal synaptic development and conformation (Rizzi et al., 2008; Paule et al., 2011; Zou et al., 2011; Lei et al., 2012). This anesthetic-induced damage might contribute to the following aging-related cerebral changes: cerebral atrophy, especially in the temporal and prefrontal regions (Hafkemeijer et al., 2014), areas involved in TMT-B performance (Zakzanis et al., 2005), and the average rate of brain volume loss (Enzinger et al., 2005).

In line with the recommendations for the nomenclature of cognitive change associated with anesthesia and surgery, the term “perioperative neurocognitive disorder” refers to “a cognitive deficit or change detected in the preoperative or post-operative period” (Evered et al., 2018). Cognitive deficits identified during preoperative assessment [hereafter referred to as preoperative neurocognitive disorders (preO-NCD)] cannot be related to the scheduled surgery and anesthesia and follow the classification of disorders as observed in the general population: mild NCD (e.g., MCI) or major NCD (e.g., dementia).

It has been estimated that preO-NCD is itself a risk factor for dementia [it converts to dementia at a rate of 10% per year (Farias et al., 2009)], delirium (Marcantonio et al., 1994; Rudolph et al., 2009; Lloyd et al., 2012), and postO-NCD (Moller et al., 1998; Lloyd et al., 2012; Paredes et al., 2016), which is particularly frequent in cardiac surgery (Linassi et al., 2022). PreO-NCD has also been associated with a strong decline in long-term behavioral functional capacity 1 year after cardiac surgery (Patron et al., 2013).

Executive function, measured via TMT-B, has been associated with 12-month postO-NCD in cardiac surgery (Messerotti Benvenuti et al., 2014); however, to the best of our knowledge, no trials have been performed to investigate the association between previous general anesthesia (pGA) and preO-NCD.

Hence, this study aims to determine the prevalence of preO-NCD in cardiac surgery and examine the role of pGA as a possible risk factor for preoperative neurocognitive disorders, measured *via* MoCA (for general cognitive function impairment) and TMT-B (for executive function impairment).

## Materials and Methods

This observational, prospective study was approved by the Ethical Committee of Azienda Ospedaliera di Padova, Italy (Protocol ID: 65408) and registered at clinicaltrials.gov (NCT04182477).

During the study period, from January 2020 to September 2021, 160 out of 168 adult patients were scheduled for cardiac surgery, coronary artery bypass grafting (CABG) or valve surgery (VS), at Padua University Hospital and provided written informed consent to participate in the study.

We excluded patients with any neurological or psychiatric disease and renal insufficiency in anamnesis. Among the neurological exclusion criteria and known strokes, patients with a history of TIA or other neurological diseases known at the time of administration of the tests were also excluded. Additionally, we excluded patients who were unable to understand the information contained in the informed consent form, patients affected by uncontrolled arterial hypertension (AHT) and diabetes mellitus (DM), patients with a history of drug and alcohol abuse, and patients without the preservation of independence in functional abilities [discovered with activity daily living (ADL) and instrumental activity daily living (IADL)] or who had significant impairment in social or occupational functioning.

**Figure 1:**
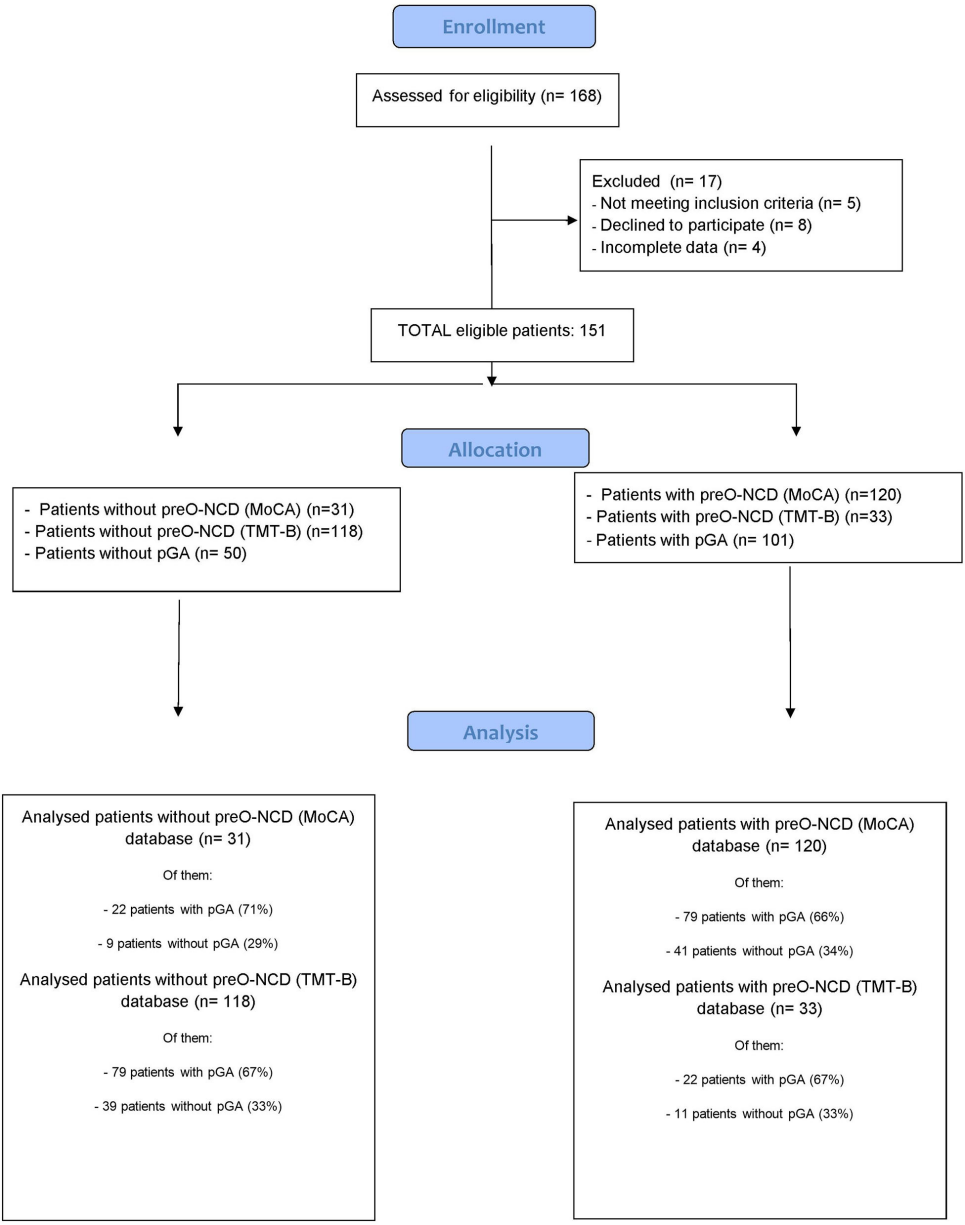
Flow chart illustrating patients' recruitment.

Therefore, of the 160 patients who gave their consent to participate, five were excluded because they did not meet the inclusion criteria, and four were excluded because of incomplete data. Therefore, data from the remaining 151 patients were analyzed (Figure 1). These patients underwent two neurocognitive tests:

- The Montreal Cognitive Assessment (MoCA) was administered to assess for general cognitive function impairment (visuospatial, executive function, naming, memory, attention, language, abstraction, and orientation).
- The Trail Making Test Part B (TMT-B) was administered to assess executive function impairment.

The tests were performed and interpreted in a silent room of the cardiac surgery unit and delivered by a neuropsychologist (ADL).

### Clinical End-Points and Variables

The primary objective was to determine the prevalence of preO-NCD in patients enrolled for cardiac surgery, analyzing both the prevalence of general cognitive function impairment [measured by a MoCA score ≤ 25 after adding 1 point if the years of education were ≤ 12 (Nasreddine et al., 2005)] and that of executive function impairment [measured by a TMT-B score ≥ 283 after the correction for age and education or the inability to complete the test in 7 min (Giovagnoli et al., 1996)]. The secondary objective was to determine whether exposure to pGAs impacts the incidence of general cognitive function and executive function impairment. The variables collected were as follows:

- Demographic variables: sex, age (continuous and dichotomised into ≥ 65 years old), weight, height, body mass index (BMI), education years (also dichotomised into ≥ 10 years), right- or left-handedness.
- Anamnestic variables (investigated during the interview with the patient and confirmed by the hospital or personal archives): age at and number of pGAs (dichotomised into ≥ 1, pediatric pGA if at < 18 years old and greater than or equal to the mean of years passed from the last pGA), diagnosis of DM, chronic obstructive pulmonary disease (COPD), obesity (BMI ≥ 30 kg·m^−2^), obstructive sleep apnoea syndrome (OSAS), thyropathy, internal carotid artery (ICA) stenosis (diagnosed *via* ecography performed within 6 months before the interview, expressed in percentage and dichotomised into ≥ 35%; Arntzen et al., 2012).
- Blood tests parameters: C-reactive protein (CPR), direct bilirubin, and total white blood cells (WBC), dichotomised into greater than our laboratory sensibility (respectively > 2.9 mg/L, > 5.1 umol/L, and > 11·10^9^/L).
- American Society of Anesthesiologists (ASA) Physical Status Classification and surgery type (VS or CABG) for enlisted patients.

### Statistical Analysis

We based the sample size calculation on an MCI prevalence of 35% (Silbert et al., 2007) for the population of cardiac surgery candidates. We considered a confidence level of 95% and a margin of error of 7.5% (Daniel and Cross, 1999). Hence, we calculated a sample of 151 patients.

We created three databases, each of which was analyzed separately: one for all the patients, one for only those who had undergone at least one administration of general anesthesia, and one for only patients who had never undergone general anesthesia administration. To evaluate the impact of timing from the last pGA on cognitive function in the elderly, we further performed a subgroup analysis on the population mean of years passed from the last pGA.

We used the Shapiro–Wilk test to test for normality. We reported continuous, normally distributed variables as mean ± standard deviation (SD) and continuous, non-normally distributed variables as median [interquartile range, IQR] and minimum to maximum. The continuous variables were dichotomised according to the previous literature's cut-off findings where available or based on values higher or lower than the mean or median value (if normally or not normally distributed, respectively). We tested for differences between the groups using the two-tailed student *t*-test or the two-tailed Mann–Whitney *U*-test for normally and non-normally distributed variables, respectively. Using the chi-square test, we reported the categorical variables as numbers (percentages) and tested for differences between the groups.

Multiple linear regression analysis was also performed to explain the relationship between one dependent variable (MoCA and TMT-B scores) and the independent variables (sex, age, obesity, education years, past general anesthesia, COPD, AHT, DM, thyropathy, CPR, WBC count, bilirubin, and surgery type). The dependent variables were dichotomised as follows: MoCA (deficitary vs. not deficitary) and TMT-B (deficitary vs. not deficitary). The independent variables were dichotomised as follows: sex (male vs. female); age [younger patients (18–64 years) vs. older patients (≥65 years)]; obesity [no obesity (BMI < 30 kg·m^−2^) vs. obesity (BMI ≥ 30 kg·m^−2^)]; education years (≥10 years vs. <10 years); WBC count; bilirubin; CPR according to the laboratory's normal range values (abnormal vs. normal value); surgery type (CABG vs. VS); and past general anesthesia (0 vs. ≥ 1). Other variables (COPD, AHT, DM, and thyropathy) were dichotomised as the presence or absence of pathology. The odds ratios (ORs) with a 95% confidence interval (CI) were determined. Multicollinearity was assessed using specific or generalized variance inflation factors. The Akaike information criterion and backward/forward stepwise regression analysis were used to choose the best model.

We used R version 3.4.0 (2017-04-21) for all statistical analyses and considered *p* < 0.05 statistically significant.

## Results

The mean (±SD) age, weight, height, and BMI of 151 patients were as follows: 66.8 ± 10.2 years old, 76.0 ± 13.4 kg, 170.2 ± 9.2 cm, and 26.2 ± 4.1 kg m^−2^, respectively. Table 1 shows the patients' general characteristics subdivided according to the variables considered.

**Table 1:** General patient, drug, and monitoring characteristics based on baseline cognitive impairment.

**Variable**	**[1.A] Continuous variables**				**[1.B] Dichotomous variables**	
	**Median**	**Mean**	**SD**	**(Min-Max)**	**Frequency (*n*)**	**Frequency (%)**
**Patient characteristics**						
Sex M/F, *n* (%)					114/37	75.5%/24.5%
Age, (yrs)	68	66.8	10.2	(35–87)		
Weight (Kg)	76	76.0	13.4	(46–110)		
Height (Cm)	170	170.2	9.2	(145–196)		
BMI, kg m^−2^	26	26.2	4.1	(16.0–39.4)		
Aging, *n* (%)					95	62.9%
Obesity, *n* (%)					23	15.2%
Education years, (yrs)	8	9.7	4.3	(3–20)		
Education yrs > 10 yrs, *n* (%)					62	41.0%
Number of pGAs	1	1.2	1.1	(0–5)		
Number of pGA ≥ 1, *n* (%)					101	66.9%
Years from the last GA	10.5	16.8	15.4	(0–52)		
Last pGA > 16 y					42	44.2%
Pediatric pGA					21	22.8%
Surgery type VS/CABG, *n* (%)					74/76	49.3%/50.7%
**Patient comorbidities**						
COPD, *n* (%)					16	10.6%
AHT, *n* (%)					84	55.6%
Diabetes, *n* (%)					42	27.8%
Thyropathy, *n* (%)					12	8.0%
OSAS					6	4.0%
Right ICA stenosis (%)	10	18.37	21.2	(0–100)		
Right ICA stenosis > 35% - *n* (%)					35	24.5%
Left ICA stenosis (%)	20	18.9	20.0	(0–70)		
Left ICA stenosis > 35% - *n* (%)					35	24.7%
Contralateral-handed ICA stenosis (%)	10	17.6	20.0	(0–100)		
Contralateral-handed ICA stenosis > 35% - *n* (%)					37	25.0%
**Patient laboratoristic data**						
CPR > 2.9 mg·L^−1^, *n* (%)					37	34.4%
WBC > 11^∧^9·L^−1^, *n* (%)					4	2.7%
D-Bilirubin > 5.1 umol·L^−1^, *n* (%)					33	25.4%
**Outcome tests**						
Deficitary MoCa					120	79.5%
Deficitary TMT-B					33	21.9%

*Data are presented as continuous variables (1. A) and dichotomic variables (1. B). Continuous variables have been described as follows: median, mean, standard deviation (SD), and min-max interval. Dichotomous variables have been described as absolute number (n) and percentage (%)*.

*M/F, male/female; yrs, years; BMI, body mass index; aging, age ≥ 65 yrs, obesity, BMI ≥30 kg·m^2^; pGA, previous general anesthesia; VS, valve surgery; CABG, coronary artery bypass grafting; COPD, chronic obstructive pulmonary disease; AHT, arterial hypertension; OSAS, obstructive sleep apnea syndrome; ICA, internal carotid artery; CPR, c-reactive protein; WBC, white blood cells count; D-bilirubin, bilirubin direct. MoCA, Montreal Cognitive Assessment; TMT-B, Trail Making Test B*.

A total of 120 patients (79.5%) had preoperative general cognitive function impairment, measured by a deficitary MoCA, whereas the remaining 33 patients (22%) had preoperative executive function impairment, measured by a deficitary TMT-B. No patient showed a deficitary TMT-B without a deficitary MoCA—i.e., no patients had deficits in executive function without general cognitive function impairment. Table 2 contains the patients' general characteristics subdivided according to the detection of preoperative general cognitive function impairment (measured by a deficitary MoCA) and executive function impairment (measured by a deficitary TMT-B).

**Table 2:** General patient, drug, and monitoring characteristics based on baseline cognitive impairment.

**Variable**	**General cognitive function**				**Executive function**			
	**Not deficitary MoCA *N* = 31 (20.5%)**	**Deficitary MoCA *N* = 120 (79.5%)**	**OR (95% CI)**	***p*-value**	**Not deficitary TMT-B *N* = 118 (78.1%)**	**Deficitary TMT-B *N* = 33 (21.9%)**	**OR (95% CI)**	***p*-value**
**Patient characteristics**								
Sex M/F, *n* (%)	21/10 (14%/7%)	93/27 (62%/18%)	1.6 (0.7–3.9)	0.3	85/33 (56%/22%)	29/4 (19%/3%)	2.8 (0.9–8.6)	0.07
Age, (yrs)	59.7 [47–72.4]	68.7 [60.2–77.2]		<0.01	65.6 [55.4–75.8]	71.2 [62.3–80.1]		<0.01
BMI, kg m^−2^	24.7 [21.3–28.1]	26.6 [22.5–30.7]		<0.05	26 [22.1–29.9]	26.9 [22.4–31.4]		0.3
Aging, *n* (%)	12 (8%)	83 (55%)	3.6 (1.6–8.1)	<0.01	71 (47%)	24(16%)	1.7 (0.7–4.1)	0.2
Obesity, *n* (%)	2 (1.3%)	21 (13.9%)	3.1 (0.7–13.9)	0.13	3 (2%)	2 (1.3%)	2.5 (0.3–15.5)	0.32
Education years, (yrs)	12.1 [8.5–15.7]	9.1 [4.9–13.3]		<0.01	10.4 [6.3–14.5]	7.2 [3.4–11]		<0.01
Education years >10 yrs, *n* (%)	20 (13%)	42 (28%)	0.3 (0.1–0.7)	<0.01	55 (36%)	7 (5%)	0.3 (0.1-0.8)	<0.05
Number of pGAs	1.4 [0–5]	1.2 [0–5]		0.53	1.3 [0–5]	1.2 [0–3]		0.9
Number of pGA ≥1, *n* (%)	22 (15%)	79 (52%)	0.8 (0.3–1.9)	0.67	79 (52%)	22 (15%)	1 (0.4-2.2)	1
Surgery type VS/CABG, *n* (%)	11/20 (7%/13%)	65/54 (43%/36%)	2.2 (0.7–3.9)	0.07	57/60 (38%/40%)	19/14 (13%/9%)	1.4 (0.6-3.1)	0.4
**Patient comorbidities**								
COPD, *n* (%)	2 (1%)	14 (9%)	1.9 (0.4–8.9)	0.5	12 (8%)	4 (3%)	1.2 (0.4–4)	0.7
AHT, *n* (%)	10 (7%)	74 (49%)	3.4 (1.5–7.8)	<0.01	62 (41%)	22 (15%)	1.8 (0.8–4)	0.2
DM, *n* (%)	4 (3%)	38 (25%)	3.1 (1–9.6)	<0.05	28 (19%)	14 (9%)	2.4 (1–5.3)	<0.05
Thyropathy, *n* (%)	1 (1%)	11 (7%)	3.0 (0.4–24.4)	0.46	10 (7%)	2 (1%)	0.7 (0.1–3.3)	1
Right ICA stenosis (%)	13.3 [0–35.9]	19.7 [0–40.4]		0.08	18.8 [0–41.1]	17 [0.2–33.8]		0.9
Right ICA stenosis > 35% - *n* (%)	4 (3%)	31 (22%)	2.2 (0.7–6.9)	0.2	29 (20%)	6 (4%)	0.6 (0.2–1.7)	0.5
Left ICA stenosis (%)	8.7 [0–25.5]	21.5 [2.8–40.2]		<0.01	18.5 [0–37.5]	20.2 [1.7–38.7]		0.7
Left ICA stenosis > 35% - n (%)	3 (2%)	32 (22%)	3.3 (0.9–11.5)	0.06	26 (18%)	9 (6%)	1.2 (0.5–3)	0.6
Contralateral-handed ICA stenosis (%)	8.7 [0–25.5]	19.7 [0–40.4]		<0.01	18.9 [0–40.4]	20.2 [0.2–40.2]		0.7
Contralateral-handed ICA stenosis >35% -: *n* (%)	3/ (2%)	34 (23%)	3.6 (1–12.8)	<0.05	27 (18%)	10 (7%)	1.4 (0.6–3.3)	0.5
**Patient blood test parameters**								
CPR >2.9 mg·L^−1^, *n* (%)	9 (8%)	28 (24%)	0.8 (0.3–2)	0.8	27 (23%)	10 (/8%)	1.3 (0.6–3.4)	0.5
WBC >11^∧^9·L^−1^, *n* (%)	0 (0%)	4 (2.7.5)	2.43 (0.1–46.4)	0.3	2 (1.33%)	2 (1.33%)	3.7 (0.5–27.6)	0.2
D-Bilirubin >5.1 umol·L^−1^, *n* (%)	5 (3.9%)	28 (21.5%)	1.6 (0.5–4.5)	0.4	28 (21.5%)	5 (3.5%)	0.5 (0.2–1.6)	0.3

*Continuous variables have been described as follows: mean [min-max]. Dichotomous variables have been described as absolute number (n) and percentage (%). Statistical significance was set at a p-value < 0.05. OR, odds ratios; 95% CI, 95% confidence interval*.

*M/F, male/female; n, number; yrs, years; BMI, body mass index; aging, age ≥ 65 yrs, obesity, BMI ≥30 kg·m^2^; pGA, previous general anesthesia; VS, valve surgery; CABG, coronary artery bypass grafting; COPD, chronic obstructive pulmonary disease; AHT, arterial hypertension; OSAS, obstructive sleep apnea syndrome; ICA, internal carotid artery; CPR, c-reactive protein; WBC, white blood cells count; D-bilirubin, bilirubin direct. MoCA, Montreal Cognitive Assessment; TMT-B, Trail Making Test B*.

Among the 101 patients with at least one pGA, 79 (78.2%) had general cognitive function impairment, whereas the remaining 22 patients (21.8%) had executive function impairment. Supplementary Table 1 contains the characteristics of the patients with at least one pGA, subdivided according to the detection of preoperative general cognitive function impairment (detected by a deficitary MoCA) and executive function impairment (measured by a deficitary TMT-B).

Among the 50 patients without any pGA, 41 (82%) had general cognitive function impairment, whereas the remaining 11 patients (22%) had executive function impairment. Supplementary Table 2 contains the characteristics of the patients without any pGA, subdivided according to the onset of preoperative general cognitive function impairment (measured by a deficitary MoCA) and executive function impairment (measured by a deficitary TMT-B).

Table 1 contains the patients' general characteristics.

Table 2 contains the patients' general characteristics subdivided according to the onset of preoperative general cognitive function impairment (measured by a deficitary MoCA) and executive function impairment (measured by a deficitary TMT-B).

Supplementary Table 1 contains the characteristics of patients with at least one pGA, subdivided according to the onset of preoperative general cognitive function impairment (measured by a deficitary MoCA) and executive function impairment (measured by a deficitary TMT-B).

Supplementary Table 2 contains the characteristics of patients without any pGA, subdivided according to the onset of preoperative general cognitive function impairment (measured by a deficitary MoCA) and executive function impairment (measured by a deficitary TMT-B).

Table 3 contains the multiple linear regression analysis.

**Table 3:** Logistic regression analysis to explain the relationship between MoCA and TMT-B tests and variables considered.

**Variables**	**MoCA**									**TMT-B**								
	**Regression model**					**Fitted regression model**				**Regression model**					**Fitted regression model**			
	**VIF**	**OR**	**L95% CI**	**U95% CI**	***p*-value**	**OR**	**L95% CI**	**U95% CI**	***p*-value**	**VIF**	**OR**	**L95% CI**	**U95% CI**	***p*-value**	**OR**	**L95% CI**	**U95% CI**	***p*-value**
**Male sex**	**1.185**	**1.67**	**0.49**	**5.68**	**0.413**					**1.281**	**3.67**	**0.90**	**14.80**	**0.067**	**3.31**	**0.95**	**11.50**	**0.059**
**Aging**	**1.268**	**2.33**	**0.74**	**7.29**	**0.146**	**2.99**	**1.01**	**8.84**	**0.047**	**1.217**	**1.51**	**0.47**	**4.82**	**0.489**				
**Obesity**	**1.033**	**3.14**	**0.33**	**29.00**	**0.314**					**1.189**	**0.74**	**0.17**	**3.23**	**0.693**				
**Education**	**1.147**	**0.33**	**0.11**	**1.00**	**0.050**	**0.35**	**0.12**	**0.98**	**0.045**	**1.126**	**0.22**	**0.06**	**0.74**	**0.014**	**0.22**	**0.07**	**0.68**	**0.008**
**pGA ≥ 1**	**1.570**	**1.14**	**0.28**	**4.50**	**0.851**					**1.743**	**1.06**	**0.284**	**3.970**	**0.928**				
**COPD**	**1.127**	**2.22**	**0.21**	**23.10**	**0.503**					**1.200**	**3.21**	**0.56**	**18.30**	**0.190**	**3.89**	**0.85**	**17.70**	**0.079**
**AHT**	**1.291**	**3.03**	**0.93**	**9.88**	**0.065**	**2.96**	**0.98**	**8.90**	**0.053**	**1.330**	**1.76**	**0.54**	**5.72**	**0.344**				
**Diabetes**	**1.191**	**1.96**	**0.43**	**8.83**	**0.379**	**2.68**	**0.65**	**10.90**	**0.169**	**1.280**	**2.70**	**0.85**	**8.59**	**0.092**	**2.75**	**0.99**	**7.65**	**0.052**
**Thyropathy**	**1.126**	**1.32**	**0.12**	**13.60**	**0.814**					**1.165**	**0.97**	**0.13**	**6.82**	**0.978**				
**CPR**	**1.081**	**0.72**	**0.23**	**2.22**	**0.574**					**1.062**	**1.50**	**0.51**	**4.35**	**0.456**				
**WBC**	**1.000**	**>100.0**	**0.00**	**inf**	**0.993**					**1.000**	**>100.0**	**0.00**	**Inf**	**0.992**				
**D-bilirubin**	**1.061**	**1.24**	**0.37**	**4.04**	**0.725**					**1.058**	**0.73**	**0.21**	**2.51**	**0.621**				
**CABG**	**1.252**	**1.58**	**0.48**	**5.16**	**0.451**					**1.212**	**0.89**	**0.30**	**2.62**	**0.840**				

*Multiple linear regression analysis was performed to explain the relationship between one dependent variable [Montreal Cognitive Assessment (MoCA score ≤ 25) and Trail-Making Test B (TMT-B score ≥ 283 s or inability to complete the test in 7 min) tests] and independent variables [sex, aging (age ≥ 65 yrs), obesity (body mass index, BMI ≥ 30 kg·m^2^), education (≥ 10 yrs), past general anesthesia (pGA), chronic obstructive pulmonary disease (COPD), arterial hypertension (AHT), diabetes, thyropathy, c-reactive protein (CPR > 2.9 mg·L^−1^), white blood cells count (WBC > 11 × 10^9^·L^−1^), bilirubin direct (D-bilirubin > 5.1 umol·L^−1^), surgery type (coronary artery bypass graft, CABG, compared to valve surgery)]. Multicollinearity was not detected using variance inflation factors (VIF). Using the Akaike Information Criterion, backward/forward stepwise regression analysis was performed to choose the best model [the fitted regression model]*.

*OR, odds ratio; L95%, lower limit of the 95% confidence interval (CI); U95%, upper limit of the 95% CI*.

*Statistical significance was set at p-value < 0.05*.

Supplementary Table 3 shows the role of age in patients with pGA performed better or worse than 16 years from the neurocognitive evaluation.

### General Cognitive Function Impairment

Advancing age (*p* < 0.01, also dichotomised into age > 65 years old, *p* < 0.01, OR 3.6), increasing BMI (*p* < 0.05), DM (*p* < 0.05, OR 3.1), AHT (*p* < 0.01, OR 3.4), increasing left ICA stenosis (*p* < 0.01), and increasing contralateral-handed ICA stenosis (*p* < 0.01, also dichotomised into > 35%, *p* < 0.05, OR 3.6) are associated with preoperative general cognitive function impairment, while increasing education years are protective (*p* < 0.01, also dichotomised into > 10 years, *p* < 0.01, OR 0.3); pGA does not seem to have any effect on general cognitive function in the general population (Table 2).

Among the patients with at least one pGA, advancing age (*p* < 0.01, also dichotomised into age > 65 years old, *p* < 0.05, OR 3.5), DM (*p* < 0.05, OR 4.3), increasing left ICA stenosis (*p* < 0.01), and increasing contralateral-handed ICA stenosis (*p* < 0.01, also dichotomised into >35%, *p* < 0.05, OR 4.5) are associated with general cognitive function impairment, whereas increasing education years (*p* < 0.01, also dichotomised into > 10 years, *p* < 0.01, OR 0.3) is protective (Supplementary Table 1). Among the patients without any pGA, advancing age (*p* = 0.01, only if considered as a continuous variable) and AHT (*p* < 0.05, OR 6.8) are associated with general cognitive function impairment (Supplementary Table 2).

When we analyzed the role of age in the subgroup of patients who underwent at least one pGA more than 16 years before cognitive evaluation, we found that age > 65 years old is associated with a general cognitive function deficit (*p* < 0.01, OR 11.4), whereas in the subgroup of patients who underwent general anesthesia less than 16 years before evaluation (55 patients), it is not (*p* = 0.4; Supplementary Table 3).

### Executive Function Deficit

Advancing age (*p* < 0.01) and DM (*p* < 0.05, OR 2.4) are associated with executive function impairment, whereas increasing education years (*p* < 0.001, also dichotomised into >10 years, *p* < 0.05, OR 0.3) is protective; pGA does not seem to have any effect on executive function in the general population (Table 2).

Among the patients with at least one pGA, advancing age (*p* < 0.01, also dichotomised into > 5 years old, *p* < 0.05, OR 4.5) and increasing WBC count (*p* < 0.05, OR 19.4) are associated with an executive function deficit, whereas increasing education years (*p* < 0.01, also dichotomised into > 10 years, *p* < 0.05, OR 0.2) is protective (Supplementary Table 1). Among the patients without any pGA, no variables were associated with executive function impairment (Supplementary Table 2).

When we analyzed the role of age in the subgroup of patients who underwent at least one pGA more than 16 years before cognitive evaluation (Supplementary Table 3), we found that age > 65 years old is associated with an executive function deficit (*p* < 0.05, OR 13.8), whereas in the subgroup of patients who underwent pGA <16 years before cognitive evaluation, it is not (*p* = 0.2).

### Logistic Regression Analysis

According to the logistic regression analysis results (Table 3), aging was associated with an increased likelihood (OR 2.99, *p* = 0.047) and education years with a decreased likelihood (OR 0.35, *p* = 0.0045) of MoCA impairment, whereas only education years were significantly associated with a decreased likelihood (OR 0.22, *p* = 0.021) of TMT-B impairment (Table 3). Past anesthesia was not shown to significantly affect MoCA and TMT-B, but a noteworthy interaction between aging and pGA was found, resulting in a synergistic effect with regard to increasing the likelihood of TMT-B impairment [OR 9.740 (1.49–63.50), *p* = 0.0174].

## Discussion

Our results show a high prevalence of preO-NCD: 79.5% of the patients present a general cognitive function deficit, whereas 22% of them present an executive function deficit. This prevalence, when compared to the incidence of MCI in the general population, is high, possibly because the international MoCA cut off of 26 (also adopted in our trial) seems to underestimate the Italian performance, resulting in global results lower than those in other countries (Bosco et al., 2017). However, our MCI prevalence is in line with recent literature findings in cardiac surgery (Scott et al., 2018; Itagaki et al., 2020), and it might increase in the next few years, which may be due to the advancing age of patients scheduled for cardiac surgery and the refinement in cardiac surgery technique, which allows surgery in frailer patients with more comorbidities. Since frailty and preO-NCD have been recently linked to delirium (Itagaki et al., 2020), delayed neurocognitive recovery, and postO-NCD—and this is related to prolonged intensive care unit (ICU), hospital stay, morbidity, and mortality (Needham et al., 2017)—a preoperative neurocognitive screening appears to be mandatory in cardiac surgery, where these post-operative complications appear to be very high (Linassi et al., 2022).

Our results about the synergic association of aging with at least one pGA in executive function impairment (measured *via* TMT-B, OR 9.7) are intriguing because neurocognitive impairment shows better relationships with pGA exposure than a general cognitive screening test such as MoCA (Messerotti Benvenuti et al., 2014). The detection of a preoperative TMT-B deficit might help track down more patients who are susceptible to anesthetics and should be strictly monitored if undergoing another form of surgery. However, future studies should elucidate the role of a pre-existing deficit in executive function and its role in postO-NCD.

### Impact of Age, Education Years, and pGA on PreO-NCD

Our linear regression analysis results show that aging is associated with general cognitive function impairment (OR 2.99), whereas it is not significant for an executive function deficit. Instead, a total of > 10 education years seems protective for both general cognitive function and executive function impairment (OR 0.35 and 0.22, respectively), confirming previous literature findings (Giovagnoli et al., 1996; Ivnik et al., 1996).

If we considered only patients with at least one pGA (Supplementary Table 1), we observed that age > 65 years old is strongly associated with executive function impairment (OR 4.5) rather than general cognitive function impairment (OR 3.5). Furthermore, our linear regression analysis confirms a synergic association between aging and pGA solely on the TMT-B (OR 9.7). This suggests that, in older patients scheduled for surgery with general anesthesia, the TMT-B should be performed together with the MoCA in the preoperative cognitive evaluation since it can better detect past neurocognitive impairment associated with anesthesia.

The fact that pGA, in the whole analyzed population, is not significant for executive function impairment (Table 2), but the association between pGA and advancing age is, whereas the association between no pGA and advancing age is not (Supplementary Tables 1, 2, respectively), suggests that anesthesia damage to executive function may be exacerbated by aging. Furthermore, the fact that, among the patients who underwent general anesthesia > 16 years before the neurocognitive evaluation, aging is significantly associated with general cognitive and executive function impairment (OR 11.4, *p* < 0.01, and OR 13.8, *p* < 0.05, respectively), while in patients with pGA <16 years before, it is not (*p* = 0.4 and *p* = 0.6, respectively, for general cognitive and executive function impairment), suggests a period of exposure-dependent damage.

Data from animal experiments strongly indicated that anesthetics commonly used in clinical practice can cause neuronal apoptosis and impair normal synaptic development and conformation, and this is particularly true in the developing brain (Rizzi et al., 2008; Paule et al., 2011; Zou et al., 2011; Lei et al., 2012). This anesthetic-induced damage adds up to the age-related loss in gray matter and to the age-related changes affecting the brain. Cerebral atrophy is particularly relevant and affects both white and gray matter, most prominent in the temporal and prefrontal regions (Hafkemeijer et al., 2014), areas involved in TMT-B performance (Zakzanis et al., 2005). Additionally, the average rate of brain volume decrease is −0.4 ± 0.29% per year and is two times faster in the elderly (age from 65 to 75 years) than in the younger population (age 50–54 years; −0.55 ± 0.29% vs −0.28 ± 0.23%; Enzinger et al., 2005).

This explains our results and strengthens the hypothesis that, in the preoperative evaluation, the TMT-B should be associated with the MoCA when evaluating every old patient who has undergone at least one pGA because it can be used to detect patients who are more susceptible to anesthetics and whose cognitive performance may worsen after another pGA. Future studies, however, should determine the association between aging, pGA, postO-NCD, and the utility of close neurocognitive follow-up in the post-operative period.

Another interpretation of the fact that the last general anesthesia administration > 16 years from the cognitive evaluation is associated with an increased risk of preO-NCD is that the conduction of general anesthesia has changed in the last few decades, with inhalational anesthesia being gradually replaced by total intravenous anesthesia (Al-Rifai and Mulvey, 2016). Some volatile anesthetics, such as halothane and sevoflurane, have been associated, in pre-clinical trials, with the increasing aggregation of Aβ amyloid (Eckenhoff et al., 2004), typical of Alzheimer's dementia (Tang and Eckenhoff, 2013), and neuronal apoptosis (Lu et al., 2010); on the contrary, it has been postulated that propofol can enhance cognitive function, reducing Aβ-induced mitochondrial dysfunction and caspase activation (Shao et al., 2014). Future studies should determine whether the last pGA administration of more than 16 years is a preO-NCD risk factor in and of itself or if it is associated with the type of anesthetic adopted.

In our results, education years is a very important protective factor in preventing preO-NCD in both the general population (*p* < 0.005, OR 0.3, for general cognitive impairment; *p* < 0.05, OR 0.3, for executive function impairment) and in patients with pGA (*p* < 0.005, OR 0.3 for general cognitive impairment and OR 0.2 for executive function impairment). Intriguingly, this protective effect appears to be diminished in patients without pGA and is not statistically significant. This can be explained from a statistical point of view (only 50 patients in the no-pGA group vs. 101 patients in the pGA group), but it may also have some neurocognitive basis.

Schooling years, in fact, alter the “cognitive reserve,” an innovative concept that explains why some people can tolerate more age- and comorbidity-related brain injury changes than others to maintain better cognitive function (Stern, 2012). Since aging and pGA have a synergistic effect on cognitive impairment, the beneficial impact of schooling on cognitive reserve may be higher in these patients than in those who did not receive these kinds of brain injuries. Future studies should, however, be conducted to confirm this theory.

### Role of Comorbidities on PreO-NCD

Our results confirm the role of multiple risk factors affecting cognitive functions, particularly DM (Cheng et al., 2012), AHT (Reitz et al., 2007), obesity (Sanderlin et al., 2017), and inflammation [i.e., higher WBC (Kao et al., 2011)]. Further, the fact that in the subgroup of patients with at least one pGA, DM is associated with higher general cognitive impairment than that in the general population (OR 4.3 vs. OR 3.1), whereas in patients without pGA, it is not significant at all, which suggests that special attention should be paid to these patients, specifically monitoring the MoCA. This result may be due to a synergistic effect between neuronal apoptosis and Aβ accumulation related to aging, anesthetic exposure, hyperglycaemia, and inflammation (Ninomiya, 2014).

In our sample, AHT is related to general cognitive function impairment (OR 3.4), but it is not related to executive function deficit, and in our subgroup analysis, it reaches statistical significance only in patients without pGA. It has been proposed that AHT impacts general cognitive function impairment through cerebrovascular injury (Pires et al., 2013), which is associated with impairment in global cognitive functions and episodic memory, typical of vascular dementia and of so-called vascular MCI (Meguro and Dodge, 2019). Thus, our results confirm that AHT and pGA have no synergistic effects, likely because they affect different brain areas and that the MoCA (and not the TMT-B) is altered in vascular MCI.

Increased BMI is significantly associated with deficitary MoCA (*p* < 0.05), but we found no significant risk associated with the dichotomised variable (BMI > 30 kg/m^2^), suggesting that this cut-off may be reconsidered. The correlation of obesity with cognitive decline is multifactorial as hypertension, dyslipidemia, and diabetes are frequently associated (Gustafson, 2006). In addition, hormones, cytokines, and inflammation mediators secreted by adipose tissue directly affect neuronal structure and functions.

TIAs are among the cardiovascular comorbidities that may potentially have an impact on cognitive function (van Rooij et al., 2016; Nicolas et al., 2020). TIAs are an exclusion criterion in this study; however, a population with misdiagnosed or underdiagnosed TIA is known to exist, and some studies [(Kessler C, Thomas KE. An examination of economic outcomes associated with misdiagnosis or undertreatment of TIA. Am J Manag Care. 2009 Jun;15(6 Suppl): S170-6. PMID: 19601692)] report that half of the patients who experience TIA are undiagnosed. Since TIAs share similar risk factors as those faced by enrolled patients, it is not possible to know the impact of undiagnosed TIAs in this study.

Our data show that left ICA stenosis and contralateral-handed ICA stenosis are associated with general cognitive dysfunction but not with executive function impairment. In the literature, two possible mechanisms of cognitive decline in patients with ICA disease have been suggested, cerebral emboli and hypoperfusion, which may lead to silent brain infarction and the loss of cerebral circulation autoregulation (Demarin et al., 2012). Intriguingly, the OR for general cognitive function impairment is higher in patients with contralateral ICA stenosis > 35%: 4.5 vs. 3.6 for left ICA stenosis. It is known that, in patients with MCI, the hippocampus, the parahippocampal gyrus, and the entorhinal cortex present more severe atrophy in the gray matter in the left hemisphere than in the right hemisphere (Thompson et al., 1998); however, future studies should elucidate the role of laterality in ICA stenosis and preO-NCD detection.

### Limitations

This study has some noteworthy limitations. First, our monocentric trial enrolled only patients scheduled for cardiac surgery, with important comorbidities that justify our higher preO-NCD incidence, but our results should be confirmed with other multi-centric studies in a non-cardiologic population.

Second, other studies with higher sample sizes should confirm our results. Further studies are also necessary to investigate the impact of pediatric pGA and the influence of different anesthetic drugs and techniques on cognitive impairment development, as well as the roles of ICA stenosis and hemisphere dominance on cognitive impairment. Another limitation is that we have not performed cerebral diagnostic radiology to detect previous undiagnosed ministrokes; further studies should elucidate their impact on preO-NCD and the association with pGA.

## Conclusion

Our study found a high incidence of preO-NCD among patients scheduled for cardiac surgery, confirming that this population should be strictly monitored for cognitive impairment, particularly before undergoing general anesthesia. Furthermore, in our linear regression analysis, advancing age is associated with general cognitive function impairment (OR 2.99) rather than executive function impairment, but there is a synergic association between aging and at least one pGA in executive function impairment (OR 9.7). This suggests that among the elderly, the TMT-B should be associated with the MoCA in preoperative cognitive evaluation.

## Data Availability Statement

The datasets presented in this study can be found in online repositories. The names of the repository/repositories and accession number(s) can be found at: https://sendgb.com/ZMQN8KRDT1u.

## Ethics Statement

The studies involving human participants were reviewed and approved by the Ethical Committee of Azienda Ospedaliera di Padova, Italy (Protocol ID: 65408). The patients/participants provided their written informed consent to participate in this study.

## Author Contributions

FL, AD, EM, LS, and AG conceived of the study, acquired, collected and analyzed data, and drafted and revised the final manuscript. FL, LS, GG, DP, PZ, and MC collected data, performed the statistical analysis, analyzed data, and revised the final manuscript. AD, EM, AG, GG, DP, PZ, and MC participated in the conceiving of the study, analyzed data, participated in the discussion of the results, and revised the manuscript. All authors contributed to the article and approved the submitted version.

## Conflict of Interest

The authors declare that the research was conducted in the absence of any commercial or financial relationships that could be construed as a potential conflict of interest.

## Publisher's Note

All claims expressed in this article are solely those of the authors and do not necessarily represent those of their affiliated organizations, or those of the publisher, the editors and the reviewers. Any product that may be evaluated in this article, or claim that may be made by its manufacturer, is not guaranteed or endorsed by the publisher.
