# Supplementary material for: Influence of Previous General Anesthesia on Cognitive Impairment: An Observational Study Among 151 Patients
Source: Front Hum Neurosci. 2022 May 16;16:810046. doi: 10.3389/fnhum.2022.810046 (PMC9148968; doi:10.3389/fnhum.2022.810046)
Supplement: Supplementary file 1 [file Data_Sheet_1.PDF]

Table S1. Patient with previous general anaesthesia, drug, and monitoring parameter characteristics

Variable	General cognitive function				Executive function			
	Not deficitary MOCA n=22 (21.8%)	Deficitary MoCA n= 79 (78.2%)	OR (95% CI)	P value	Not deficitary TMT-B n= 79 (78.2%)	Deficitary TMT-B n= 22 (21.8%)	OR (95%CI)	P value
**Patient characteristics**								
Sex (M/F)- n (%)	14/8 (14%/7.9%))	61/18 (60%/17.8%)	1.9 (0.7-5.3)	0.3	57/22 (56%/22%)	18/4 (18%/4%)	1.7 (0.5-5.7)	0.4
Age (years old)	61.2 [48.5-73.9]	69.3 [60.7-77.9]		<0.01	66 [55.7-76.3]	73.1 [65.7-80.5]		<0.01
BMI (kg m-2)	24.7 [21.1-28.3]	26.2 [22.1-30.3]		0.1	25.6 [21.7-29.5]	26.7 [22.1-31.3]		0.25
Aging	9 (9%)	56 (55%)	3.5 (1.3-9.3)	<0.05	43 (46%)	13 (19%)	4.5 (1.2-16.6)	<0.05
Obesity BMI>30, n (%)	2 (2%)	13 (12.9%)	2.0 (0.4-9.5)	0.4	9 (8.9%)	6 (5.9%)	2.9 (0.9-9.4)	0.06
Education years (yrs)	12.3 [8.6-16]	8.8 [4.6-13]		<0.01	10.4 [6.3-14.5]	6.8 [2.8-10.8]		<0.01
Education years >10 yrs - n (%)	14 (14%)	26 (26%)	0.3 (0.1-0.8)	<0.01	33 (36%)	4 (4%)	0.2 (0.08-0.9)	<0.05
number of pGAs	1.9 [0.8-3]	1.8 [1.1-2.7]		1	1.9 [1-2.8]	1.8 [1-2.6]		0.9
Years from last GA	18 [1.9-34.1]	16.5 [1.2-31.8]		0.8	15.8 [0.9-30.7]	20.5 [3.5-37.5]		0.2
Last pGA >16 ya - n (%)	10 (11%)	32 (34%)	0.7 (0.3-2)	0.6	31 (33%)	11 (12%)	1.5 (0.6-4)	0.5
Age at first GA (years old)	33 [13-53]	43.2 [24.8-61.6]		0.06	32 [13.1-50.9]	46 [24.8-67.2]		0.7
Paediatric pGA - n (%)	5 (5%)	16 (17%)	0.9 (0.3-2.9)	1	15 (16%)	6 (7%)	1.8 (0.6-5.5)	0.4
Surgery type VS/CABG: n (%)	8/14 (8%/14%)	41/38 (41%/38%)	1.9 (0.7-5)	0.2	35/44 (35%/44%)	14/8 (14%/8%)	2.2 (0.8-5.8)	0.2
**Patient comorbidities**								
COPD, n (%)	2 (2%)	10 (10%)	1.4 (0.3-7.1)	1	8 (8%)	4 (4%)	1.9 (0.5-7.3)	0.3
AHT, n (%)	8 (8%)	47 (47%)	2.5 (0.9-6.8)	0.09	40 (40%)	15 (15%)	2 (0.8-5.7)	0.2
DM, n (%)	2 (2%)	24 (24%)	4.3 (0.9-20.1)	<0.05	18 (18%)	8 (8%)	1.9 (0.7-5.3)	0.3
Thyropathy, n (%)	0 (0%)	7 (7%)	4.6 (0.3-84)	0.3	6 (6%)	1 (1%)	0.6 (0.06-5)	1
Right ICA stenosis (%)	10 [0-26.1]	19.4 [0-40.3]		0.07	17.3 [0-38.7]	17.7 [0.7-34.7]		0.7

Right ICA stenosis > 35% n (%)	2 (2%)	19 (20%)	3.1 (0.7-15)	0.2	17 (18%)	4 (4%)	0.8 (0.2-2.6)	0.8
Left ICA stenosis (%)	8.6 [0-24.2]	22.1 [2.6-41.6]		<0.01	18.2 [0-37.4]	22.7 [2.2-43.2]		0.4
Left ICA stenosis >35% - Y/N: n (%)	2 (2%)	23 (25%)	4.1 (0.9-19)	0.08	17 (18%)	8 (9%)	1.9 (0.7-5.6)	0.3
Contralateral-handed ICA stenosis (%)	8.6 [0-24.2]	23.1 [1.6-44.6]		<0.01	15 [0-36.3]	22 [1.9-42.1]		0.5
Contralateral-handed ICA stenosis >35% -: n (%)	2 (2%)	25 (26%)	4.5 (0.9-21)	<0.05	18 (18%)	9 (9%)	2.2 (0.8-6)	0.2
**Patient laboratoristic data**								
CPR > 2.9 mg/L. - Y/N: n (%)	9 (12%)	16 (21%)	0.4 (0.1-1.2)	0.2	19 (25%)	6 (8%)	1.3 (0.4-4.1)	0.8
WBC >11^9·L^{-1}, n (%)	0 (0%)	2 (2%)	1.5 (0.1-31.4)	0.5	0 (0%)	2 (2%)	19.4 (0.9-420)	<0.05
D-Bilirubin >5.1 umol·L^{-1}, n (%)	4 (4.7%)	18 (21%)	1.4 (0.4-4.7)	0.6	18 (20.9%)	4 (4.7%)	0.7 (0.2-2.5)	0.6

OR: odds ratio; 95%CI: 95% confidence interval; M/F: male/female; n: number; yrs: years; BMI: body mass index; aging: age ≥ 65 yrs, obesity: BMI ≥30 kg·m^2; pGA: previous general anaesthesia; VS: valve surgery; CABG: coronary artery bypass grafting; COPD: chronic obstructive pulmonary disease; AHT: arterial hypertension; OSAS: Obstructive Sleep Apnea Syndrome; ICA: Internal Carotid Artery; CPR: c-reactive protein; WBC: white blood cells count; D-bilirubin: bilirubin direct. MoCA: Montreal Cognitive Assessment; TMT-B: Trail Making Test B; Continuous variables have been described with: mean [min-max]; Dichotomous variables have been described with absolute frequency and relative frequency. ORs for dichotomous variables have been described with absolute number and 95% confidence interval. Statistical significance was set at p-value <0.05

Table S2. Patient without previous general anaesthesia, drug, and monitoring parameter characteristics

Variable	General cognitive function				Executive function			
	Not deficitary MOCA 9 (18%)	Deficitary MoCA n= 41 (82%)	OR (95% CI)	P value	Not deficitary TMT-B n=39 (78%)	Deficitary TMT-B n=11 (22%)	OR (95%CI)	P value
**Patient characteristics**								
Sex (M/F): n (%)	7/2 (14%/4%)	32/9 (64%/18%)	1.0 (0.2-5.8)	0.9	28/11 (56%/22%)	11/0 (22%/0%)	9.3 (0.5-171)	0.06
Age (years old)	56 [43-80]	67.4 [46-80]		0.01	64.8 [43-80]	67.2 [46-80]		0.5
BMI (kg m-2)	24.9 [19.4-29.7]	27.4 [20.7-39.4]		0.09	26.9 [19.4-35.1]	27.1 [20.7-39.4]		0.9
Aging	3 (6%)	27 (54%)	3.9 (0.8-17.8)	0.07	25 (50%)	5 (10%)	0.5 (0.1-1.8)	0.3
Obesity, n (%)	0 (0%)	8 (16%)	4.8 (0.3-91.4)	0.15	7 (14%)	1 (2%)	0.5 (0.05-4.2)	0.5
Education years (yrs)	11.6 [5-17]	9.7 [5-20]		0.24	10.6 [5-20]	8.0 [5-13]		0.08
Education years >10 yrs -: n (%)	7 (14%)	17 (35%)	0.2 (0.1-1.1)	0.05	19 (38%)	3 (6%)	0.4 (0.1-1.7)	0.2
Surgery type VS/CABG: n (%)	6/3 (12%/6%)	17/24 (34%/48%)	2.8 (0.6-12.9)	0.2	17/22 (34%/44%)	6/5 (12%/10%)	0.6 (0.2-2.5)	0.5
**Patient comorbidities**								
COPD, n (%)	0 (0%)	4 (8%)	2.3 (0.1-46.1)	0.3	4 (8%)	0 (0%)	0.3 (0.02-6.9)	0.3
AHT, n (%)	2 (4%)	27 (54%)	6.8 (1.2-36.9)	<0,05	22 (44%)	7 (14%)	1.4 (0.3-5.4)	0.7
Diabetes, n (%)	2 (4%)	14 (28%)	1.8 (0.3-9.9)	0.5	10 (20%)	6 (12%)	3.5 (0.9-13.9)	0.07
Thyropathy, n (%)	1 (2%)	4 (8%)	0.9 (0.1-8.8)	0.9	4 (8%)	1 82%)	0.9 (0.1-8.7)	0.9
Right ICA stenosis (%)	21.1 [0-100]	20.1 [0-60]		0.9	21.6 [0-100]	15.5 [0-40]		0.4
Right ICA stenosis > 35% n (%)	2 (4 %)	12 (24.5%)	1.2 (0.2-7.0)	0.8	12 (24.5%)	2 (4%)	0.5 (0.1-2.6)	0.4
Left ICA stenosis (%)	8.9 [0-60]	20.2 [0-50]		0.09	19.1 [0-60]	15 [0-50]		0.5

Left ICA stenosis >35% - n (%)	1 (2%)	9 (18.4%)	2.0 (0.2-18.2)	0.5	9 (18.4%)	1 (2%)	0.3 (0.03-2.9)	0.3
Contralateral-handed ICA stenosis (%)	8.9 [0-60]	19.8 [0-50]		0.1	18.6 [0-60]	15.0 [0-50]		0.6
Contralateral-handed ICA stenosis >35% - n (%)	1 (2%)	9 (18%)	2.3 (0.2-20.4)	0.5	9 (18%)	1 (2%)	0.3 (0.04-3.0)	0.3
**Patient laboratoristic data**								
CPR > 2.9 mg/L. - : n (%)	0 (0%)	12 (23.3%)	8.3 (0.4-158.4)	0.06	8 (19.5%)	4 (9.7%)	1.6 (0.4-6.8)	0.5
WBC >11^9·L^{-1}, n (%)	0 (0%)	2 (4&)	1.2 (0.05-27.2)	0.5	2 (4%)	0 (0%)	0.7 (0.03-14.6)	0.4
D-Bilirubin >5.1 umol·L^{-1}, n (%)	1 (2.3%)	10 (22.7%)	2.2 (0.2-20.8)	0.5	10 (22.7%)	1 (2.3%)	0.3 (0.03- 2.4)	0.2

OR: odds ratio; 95%CI: 95% confidence interval; M/F: male/female; n: number; yrs: years; BMI: body mass index; aging: age ≥ 65 yrs, obesity: BMI ≥30 kg·m^2; pGA: previous general anaesthesia; VS: valve surgery; CABG: coronary artery bypass grafting; COPD: chronic obstructive pulmonary disease; AHT: arterial hypertension; OSAS: Obstructive Sleep Apnea Syndrome*;* ICA: Internal Carotid Artery; CPR: c-reactive protein; WBC: white blood cells count; D-bilirubin: bilirubin direct. MoCA: Montreal Cognitive Assessment; TMT-B: Trail Making Test B; Continuous variables have been described with: mean [min-max]; Dichotomous variables have been described with absolute frequency and relative frequency. ORs for dichotomous variables have been described with absolute number and 95% confidence interval. Statistical significance was set at p-value <0.

Table S3. Role of age in patients with pGA performed > or <16 years from neurocognitive evaluation and role of age in patients with pediatric or not pGA.

Variable	General cognitive function				Executive function			
	Not deficitary MOCA	Deficitary MoCA	OR (95% CI)	P value	Not deficitary TMT-B	Deficitary TMT-B	OR (95%CI)	P value
**pGA > 16 years from neurocognitive evaluation, n (%)**	10 (10.5%)	32 (33.0%)	0.7 (0.3-2)	0.5	31 (32.6%)	11 (11.6%)	1.5 (0.6-4.0)	0.4
Aging, n (%)	2 (4.2%)	25 (52.1 %)	11.4 (2.1-60.7)	<0.01	16 (59.2%)	11 (40.7%)	13.8 (1.6-118.1)	<0.05
Age, (yrs)	56.7 [43-80]	68.7 [52-82]	/	<0.01	63.3 [43-82]	72.9 [61-82]	/	<0.05*
**pGA < 16 years from neurocognitive evaluation, n (%)**	10 (10.6%)	45 (47.9%)	1.5 (0.6-4.2)	0.4	44 (46.3%)	11 (11.6%)	0.8 (0.3-2.0)	0.6
Aging, n (%)	7 (11.5%)	35 (57.4%)	1.7 (0.5-6.7)	0.4	32 (52.6%)	10 (16.4%)	2.7 (0.5-13.5)	0.2
Age, (yrs)	63.1[35-79]	70.2 [49-87]	/	<0.05*	67.5 [35-86]	74.3 [61-87]	/	<0.05

Continuous variables have been described as follows: mean [min-max]. Dichotomous variables have been described as absolute number (n) and percentage (%) with relative frequency calculated as the total of patients with previous general anesthesia of the relative subgroups). OR: odds ratio; 95%CI: 95% confidence interval; aging: age ≥ 65 yrs; pGA: previous general anaesthesia; MoCA: Montreal Cognitive Assessment; TMT-B: Trail Making Test B. Statistical significance was set at p-value <0.05. (*) Levene's test is significant (p < .05), suggesting a violation of the equal variance assumption.
